# Meeting Review: 2018 International Workshop on Structure and Function of the Lentiviral gp41 Cytoplasmic Tail

**DOI:** 10.3390/v10110613

**Published:** 2018-11-07

**Authors:** Melissa V. Fernandez, Eric O. Freed

**Affiliations:** HIV Dynamics and Replication Program, Center for Cancer Research, National Cancer Institute, Frederick, MD 21702, USA; Melissa.Fernandez2@nih.gov

**Keywords:** gp41, workshop, envelope, glycoprotein, transmission, incorporation, trafficking, cytoplasmic tail, lentivirus, simian immunodeficiency virus (SIV), murine leukemia virus (MLV), HIV

## Abstract

Recent developments in defining the role of the lentiviral envelope glycoprotein (Env) cytoplasmic tail (CT) in Env trafficking and incorporation into virus particles have advanced our understanding of viral replication and transmission. To stimulate additional progress in this field, the two-day International Workshop on Structure and Function of the Lentiviral gp41 Cytoplasmic Tail, co-organized by Eric Freed and James Hoxie, was held at the National Cancer Institute in Frederick, MD (26–27 April 2018). The meeting served to bring together experts focused on the role of gp41 in HIV replication and to discuss the emerging mechanisms of CT-dependent trafficking, Env conformation and structure, host protein interaction, incorporation, and viral transmission. The conference was organized around the following three main hot topics in gp41 research: the role of host factors in CT-dependent Env incorporation, Env structure, and CT-mediated trafficking and transmission. This review highlights important topics and the advances in gp41 research that were discussed during the conference.

## 1. Introduction

Primate lentiviral envelope glycoprotein (Env) glycoproteins play key roles in viral replication. Yet, an incomplete understanding of Env function during viral assembly and maturation impedes treatment and vaccine design against HIV infection. The fundamental role of Env is to mediate viral entry into uninfected cells. Viral transmission can occur through cell-free infection or via the cell-to-cell transfer of virus particles at points of contact between infected and uninfected cells, known as the virological synapse (VS).

**Figure 1:**
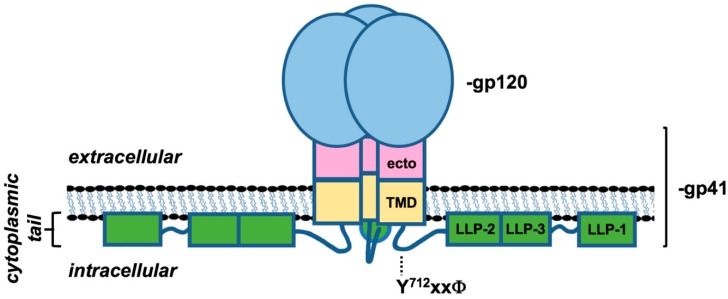
Schematic representation of the HIV envelope glycoprotein (Env) trimer of dimers (adapted from Tedbury and Freed [1]). The gp120 subunit is in pink and the three domains of the gp41 subunit are represented in pink, yellow, and green. The plasma membrane is indicated in black with the transmembrane domain (TMD) of gp41 spanning the membrane, bridging the extracellular gp120 subunit and the intracellular cytoplasmic tail (CT). The location of the conserved endocytosis motif, Y^712^xxΦ, is indicated with a dashed line. The location of the three lentivirus lytic peptides (LLP) in the CT are indicated along the CT.

Primate lentiviral Env glycoproteins are expressed from the *env* gene as an unprocessed, immature precursor, gp160. The mature HIV Env glycoprotein is composed of two subunits—the surface subunit, gp120, and the transmembrane subunit, gp41 (Figure 1). The two subunits are non-covalently linked to form a gp120:gp41 heterodimer. Three gp120:gp41 dimers form the mature, functional Env glycoprotein complex present on the surface of infected cells and virus particles. The gp120 subunit mediates receptor/co-receptor binding; gp41 comprises three domains, namely: an extracellular ectodomain that tethers gp120 to the cell surface and is the principle driver of membrane fusion, a transmembrane domain (TMD) that anchors Env in the lipid bilayer, and the cytoplasmic tail (CT) that regulates a number of aspects of Env function.

The role of the matrix (MA) in Env incorporation has been long appreciated, yet the precise mechanism of the MA–Env interaction is unknown. Mutations in MA that block Env incorporation are overcome by Env CT truncation. Compensatory mutations in MA that rescue Env incorporation have been identified, further underscoring the important role of MA in Env incorporation. Several models of Env incorporation into the assembling Gag lattice have been proposed [1]. Different cell lines and types may be differentially dominated by one model of Env incorporation, complicating the understanding of Env incorporation. Factors such as the Env recycling kinetics, Env expression levels, lipid raft content, and CT structure play a role in Env targeting to the assembling Gag lattice.

The CTs of HIV-1, HIV-2, and SIV gp41 are typically ~150 amino acids in length, although naturally truncated forms can arise, particularly for HIV-2 and SIV (see below). Lentiviral gp41 CTs contain a variety of conserved motifs and structures. The N-terminus of the CT contains a disordered loop with a highly conserved endocytosis motif (Y^712^xxΦ). The remainder of the CT comprises three alpha helical motifs known as the lentiviral lytic peptides (LLP-2, LLP-3, and LLP-1), which are predicted to be tightly associated with the inner leaflet of the plasma membrane or the viral lipid bilayer (Figure 1). The CT is dispensable for binding the CD4 and co-receptor (CCR5 or CXCR4), but is essential for retrograde Env trafficking, Env incorporation into nascent virions, and spreading infection in physiologically relevant cell types. The CT domain also modulates the Env structure and fusogenicity.

While some basic aspects of the gp41 CT function are known, the precise mechanisms by which the CT influences Env trafficking and recycling, Env incorporation, Env conformation, VS formation, cell-to-cell transmission, and interaction of gp41 with host cell factors are largely uncharacterized. An improved understanding of the CT function would facilitate the development of novel anti-viral therapy and would enhance vaccine design strategies. These topics were briefly introduced by Eric Freed at the beginning of the workshop, and recent progress in the field is described below.

## 2. Host Factors, Conformation, and Function

Paul Spearman (Children’s Cincinnati Hospital Medical Center) opened the session on host factors by discussing his work on the roles of Rab14- and Rab11-family interacting protein 1C (Rab11-FIP1C) in Env recycling and incorporation. Spearman’s earlier work had demonstrated that the knockdown (KD) of the endosomal proteins FIP1C and Rab14 resulted in a significant reduction in Env incorporation [2]. The gp41 CT, and more specifically an aromatic motif (YW_795_) in the LLP-3 domain of the CT, was found to be essential for the Env-induced relocalization of FIP1C from the endosomal recycling compartment (ERC) to the cell periphery. These observations suggested that this aromatic motif may be involved in the direct or indirect interactions between the gp41 CT and FIP1C [3]. Full-length HIV-1 Env was shown to be retained in an aberrant ERC induced by a dominant-negative FIP1C mutant, and the Env incorporation was impaired under these conditions. The truncation of the gp41 CT, or the mutation of the Y^712^xxΦ or YW_795_ motifs in the CT, relieved the Env retention induced by the dominant-negative FIP1C, and restored the Env incorporation. These results are consistent with the hypothesis that Env is internalized and sorted through the ERC prior to its incorporation into virions [4]. The SIVmac239 Env was not retained in the aberrant ERC induced by dominant-negative FIP1C [4]. Spearman ended by presenting preliminary data indicating that, like SIVmac239 Env, the Env glycoproteins of some strains of HIV-1 are not retained in the ERC upon expression of dominant-negative FIP1C. These results suggest that the utilization of FIP1C is HIV-1 strain-dependent, or that some strains of HIV-1 use a non-ERC pathway for Env incorporation.

Melissa Fernandez (HIV Dynamics and Replication Program, National Cancer Institute, from Eric Freed’s group) continued by discussing the role of aromatic residues in, and adjacent to, the FIP1C relocalization motif, YW_795_. The non-conservative substitution of a single aromatic residue resulted in mild defects in replication kinetics, while the substitution of two aromatics caused severe replication defects. Similarly, the mutation of the conserved Y_795_ in a clade C transmitter/founder (T/F) virus abolished the viral replication. Fernandez also described work related to the observation, made many years ago in the Freed lab, that the truncation of the gp41 CT blocks HIV-1 replication in primary T-cells and most T-cell lines, whereas the human T-lymphotropic leukemia virus (HTLV-1)-transformed MT-4 T-cell line supports the robust replication of CT-truncated HIV-1 [5]. Fernandez found that MT-4 cells are FIP1C-deficient, yet are capable of incorporating Env containing either full-length, truncated, or mutant CTs. The ability of MT-4 to overcome its FIP1C deficiency with respect to Env incorporation may be due to an enhanced HIV protein production relative to other T-cell lines. Fernandez concluded by discussing her plans to determine whether the HTLV-I Tax expression in MT-4 cells could drive enhanced HIV protein production and CT-independent Env incorporation.

The successful dissemination of HIV-1 in infected individuals depends on the efficient transmission of viral particles from infected to uninfected cells, without excessive syncytium formation. Markus Thali (University of Vermont) continued the session by presenting his work on the regulation of cell–cell fusion in HIV-1 transfer across the VS. The Thali group previously reported that Env clusters with Gag on the surface of virus-producing cells, and this Gag-mediated clustering requires the gp41 CT [6]. The interaction of Gag with the gp41 CT may suppress the Env-mediated cell–cell fusion [6]. Cellular factors, including the tetraspanins CD63 and CD9, EWI-2, and ezrin, also repress the formation of syncytia and facilitate fusion-less encounters between the producer and target cells at the VS [7,8,9,10]. In 3D extracellular matrix hydrogels, small T-cell syncytia can transfer the virus to uninfected T-cells in the absence of a further cell–cell fusion [11]. A central theme of this work is that Env-induced membrane fusion is regulated by both viral and cellular factors, runaway fusion is disadvantageous for the virus, and the gp41 CT is a major contributor to the regulation of Env-mediated fusion activity.

Benjamin Chen (Icahn School of Medicine at Mount Sinai) provided insights into the function of the membrane-proximal Y^712^xxΦ endocytosis motif by exploring the role of this motif in neutralization, by broadly neutralizing the antibodies (bNAbs) capable of recognizing epitopes shared by many HIV-1 isolates [12]. The Env from two lab-adapted molecular clones, NL4-3 and JR-FL, and two subtype B transmitted/founder (T/F) Envs, QH0692 and RHPA, were selected for detailed study. These Envs can exhibit incomplete neutralization by HIV-Ig against a cell-associated virus, but the cell-free form was fully inhibited. Chen also found patient-specific differences in the neutralization efficiency against cell-associated and cell-free Env. Furthermore, the CT was found to play a crucial role in viral resistance to bNAbs during cell-to-cell transmission. The disruption of the membrane-proximal Y^712^xxΦ motif enhanced neutralization by bNAbs, indicating that the endocytosis motif also modulates the presentation of neutralizing epitopes on the surface of the infected cells, and thus also regulates the conformation of the ectodomain. A Y^712^xxΦ mutant increased the levels of Env at the VS, but reduced virus transmission. These findings indicate that the virus can escape bNAb-mediated neutralization through cell-to-cell viral transmission, and that the gp41 CT, and specifically the Y^712^xxΦ motif, modulates the exposure of neutralizing epitopes. Interestingly, a recent study demonstrated that, in the SIV system, the truncation of the gp41 CT can decrease sensitivity of NAbs [13].

Akira Ono (University of Michigan Medical School) ended the session with a discussion of polarized HIV-1 Gag targeting during virion assembly, and the role of fibroblastic reticular cells (FRCs) in HIV-1 dissemination. Polarized T-cells are characterized by a rear-end protrusion, known as the uropod, that functions in forming the VS. During assembly, Gag co-clusters with PSGL-1, CD43, and CD44 on uropod-directed microdomains (UDMs) in a MA-dependent manner, causing a re-organization of the UDM to exclude ICAM-1 and ICAM-3 [14]. Furthermore, the specific association between Gag and the UDM is dependent on the highly basic region in MA [15]. Ono also described some recently published work demonstrating that FRCs, which are stromal cells in lymph nodes, capture HIV-1 particles and facilitate their productive transfer to T-cells, a process known as “trans-infection” [16]. A key component of this process is the incorporation of the uropod marker CD44 into HIV-1 virions; this incorporated the CD44 binds of hyaluronan, which in turn binds CD44 on the surface of the FRCs, allowing for viral capture.

## 3. Structure

Several lines of evidence suggest that an interaction between the gp41 CT and the MA domain of Gag facilitates the Env incorporation into budding HIV-1 particles. Chris Aiken (Vanderbilt University) began the session on structure by discussing the determinants in the gp41 CT involved in the gp41-MA interaction. Early work from Aiken’s lab demonstrated that gp41 could be retained on immature HIV-1 particles, following the detergent-mediated removal of the viral lipid bilayer. This detergent-resistant association of Env with the particle required the gp41 CT, suggesting that the CT remained associated with Gag, even after removal of the viral lipid envelope [17]. Aiken also described new unpublished work performed in collaboration with Charles Sanders, in which the binding of purified recombinant gp41 CT to Gag virus-like particles (VLPs) was measured in vitro. A mutation, 62QR, reported by the Freed lab to rescue Env incorporation defects [18], significantly increased the binding of the gp41 CT protein fragment to Gag VLPs; conversely, the d8 deletion mutation, which removes five amino acids from the gp41 CT and impairs Env incorporation [19], reduces CT–VLP binding. These results suggest that the binding assay may be physiologically relevant and thus useful in the study of gp41 CT–MA interactions. 

The structure of the gp41 CT has been enigmatic and is an area of great interest to the field. Recently, Jamil Saad (University of Alabama at Birmingham) used NMR methods to solve the first structure of the CT [20]. The protein was produced via recombinant techniques and the structure was determined in a micellar solution. In agreement with the working model for the general structure of the CT, the data revealed that the N-terminal 45 residues of the CT are unstructured and are not associated with the membrane. The C-terminal 105 residues, however, form three clearly defined membrane-bound α-helices (the LLPs discussed above). The LLPs vary in their degree of membrane penetration, hydrophobicity, charge, the presence of clustered aromatic residues, and π–π and cation–π interactions. Many of these features are well conserved, likely because of their requirement in maintaining the structure of the CT. The structure presented by Saad provides insight into prior mutagenesis studies and suggests new directions for investigation. Saad ended his talk by discussing the next step in this work—thecharacterization of gp41 CT binding to MA trimers using membrane nanodisc technology.

Walther Mothes (Yale University) described his efforts to use single-molecule Forster resonance energy transfer (smFRET) to probe the conformation of HIV-1 Env [21,22]. This work revealed that native Env trimers exist in three conformational states, namely: a pre-triggered state (state 1), an intermediate state (state 2), and a state in which each monomer in the trimer is CD4-bound (state 3). No major effect of CT deletion on Env conformation was observed using this approach. Mothes also presented previous evidence establishing a role for the CT of the murine leukemia virus Env protein in polarized viral assembly, and for the co-localization of Env and MA at the VS [23,24,25].

Kashif Sadiq (Heidelberg Institute for Theoretical Studies) focused on molecular modeling and molecular dynamics simulations by describing the initial steps to use atomistic, course-grained, and ultra-course-grained molecular dynamics simulations in order to understand the reaction kinetics of retroviral particle maturation and the coupling of maturation and Env diffusion. Sadiq’s research aims to model the gp41 CT and its interactions with Gag and the membrane. Although these approaches are very computationally demanding, they have the potential to significantly advance our understanding of Env and Gag behavior on HIV-1 particles, and to reveal the transient structures that are difficult to characterize experimentally.

Bing Chen (Harvard Medical School) asked the question, “what does a native/functional trimer look like?” The answer to this question is not straightforward—heterogeneity among the published Env structures makes it difficult to define the structure of a “functional” Env trimer. Chen has investigated the contribution of the gp41 CT to the antigenic properties of gp160 trimers in order to gain insight into HIV vaccine development. The truncation of the CT results in a compact gp140 that maintains most of the antigenic properties of native (gp120/gp41) Env trimers, but in which binding by trimer-specific antibodies is reduced and binding by non-neutralizing antibodies is increased, relative to native, non-truncated Env [26,27]. Chen also discussed his work aimed at a better understanding how the gp41 TMD influences the gp120 structure. An NMR analysis of HIV-1 Env in membrane bicelles revealed that the TMD is a tight three α-helical coiled-coil [28]. Mutations in the TMD were found to influence the Env antibody reactivity, demonstrating a role for the TMD in modulating the Env conformation [29]. Finally, unpublished work analyzing the structure of a fragment of Env, which included the TMD and a portion of the CT, demonstrated that LLP-3 packs against the membrane and the TMD.

The work presented by Prabuddha Sengupta (Howard Hughes Medical Institute) uses total internal reflection fluorescence (TIRF) and super-resolution microscopy to probe how host cell membrane proteins are sorted into retroviral particles. Using cell lines stably expressing fluorescent probes, he observed that the proteins that partition into the liquid-ordered (L_O_) phase co-localize with HIV Gag, whereas the proteins in the liquid-disordered (L_D_) phase do not. The palmitylation of MLV Env is required for its L_O_ partitioning and for robust recruitment to HIV assembly sites. These findings support the role of lipid-phase partitioning in regulating how proteins are incorporated into retroviral particles. Sengupta measured the kinetics of Gag assembly and the role of phase-separation and membrane curvature in protein redistribution. Proteins co-expressed with Gag were redistributed at different times during assembly; for example, both MLV Env and GPI-anchored proteins were recruited throughout the Gag assembly process (although GPI-anchored recruitment started a little later). Tetherin, in contrast, was recruited to the Gag assembly sites at the end of the assembly process in a manner that was regulated by its membrane anchors. Transmembrane proteins were excluded from the assembly site towards the end of the particle assembly. Sengupta employed an HIV-1 CA mutant that assembles in flat patches of Gag in order to show that membrane curvature appears to regulate late-stage protein recruitment to, or exclusion from, Gag assembly sites. These results are relevant not only for retroviral assembly, but also for protein sorting in transport vesicles and exosomes.

Like Bing Chen (see above), Joseph Sodroski (Harvard Medical School) discussed the role of the gp41 CT on Env conformation [30]. Using the HIV-1_AD8_ isolate, he showed that CT truncation minimally influences neutralization by a panel of ABS. Full-length and CT-truncated Env were also similarly triggered by soluble CD4. Together with Walther Mothes, Sodroski compared the conformational states of uncleaved gp160 and gp120 by using single-molecule fluorescence resonance energy transfer (smFRET) and cryo-electron microscopy (cryo-EM). This analysis indicated that the conformation of uncleaved gp160 is more heterogeneous than that of gp120. Native Env trimers [gp120/gp41]_3_ preferentially occupy state 1, and resist the binding of bNAbs that recognize state 2 and state 3 conformations, whereas the conformation of uncleaved gp160 samples states 1, 2, and 3 [31]. The cryo-EM analysis of uncleaved Env at a resolution of 6–8 Å indicated the presence of two states, one of which was state 2-like, the other of which resembled virion Env. These findings have potential clinical relevance, because uncleaved Env is presented to the immune system and may divert the antibody response away from the state 1 conformation, which might elicit antibodies that more effectively target functional Env trimers.

Eric Barklis (Oregon Health and Science University) discussed a long-standing question in the field—does Env directly bind MA, and if so, why are so few Env trimers packaged onto HIV-1 particles? To address these questions, Barklis found that the 62QR HIV-1 MA mutant, reported by the Freed lab to rescue Env incorporation defects [18], forms trimers more efficiently than WT MA in a UV-crosslinking assay, and is bound more efficiently than WT MA to GST-coupled gp41 CT [32]. The presence of RNA reduced binding between MA and the gp41 CT, perhaps by diminishing MA trimerization, and inositol hexakisphosphate (IP_6_) increased MA binding to the gp41 CT, perhaps by increasing the MA trimerization. This result supports a link between the formation of MA trimers and the binding of MA to the gp41 CT.

## 4. Env Trafficking and Incorporation

Schuyler Van Engelenburg (University of Denver) started the session by discussing his recently published findings involving the use of the superresolution microscopy approach, known as interferometric photo-activated localization microscopy (iPALM), to resolve the distribution of Env on cell-associated particles in 3D [33]. Because of the sparsity of Env on individual particles, Van Engelenburg derived statistical power from the single-particle averaging of hundreds of aligned individual HIV-1 assembly sites to determine the probability density of Env at the crown of the budding particle, relative to the particle equator. The investigators found that, in a manner that was dependent on both the cell type and the gp41 CT, Env tends to be concentrated near the necks of cell-associated particles. The study provided evidence that a CT intragenic deletion mutant, called d8 [19], blocks incorporation by one of two mechanisms, namely: trapping Env in intracellular compartments by preventing its interaction with endosomal recycling machinery, and/or steric hinderance with the Gag lattice, excluding Env from the assembling particle [33]. Their findings support a model wherein WT Env is internalized in a CT-dependent manner, followed by limited recycling of Env back to the PM, which is temporally delayed relative to the Gag lattice assembly, thus resulting in the localization of small numbers of Env trimers, preferentially near the neck of the assembling particle.

James A. Hoxie (University of Pennsylvania) and Mark Marsh (University College London) presented two inter-connected talks, in which they extended the tissue culture experimentation to an in vivo model, the SIVmac infection of macaques. In pathogenic models of SIV and HIV infection, CD4+ cells in the gut are often decimated rapidly, leading to a loss of the epithelial barrier, creating a host with intense systemic immune activation. Thinking of pathogenesis as a triangle of time, location, and kinetics raised the possibility that, if one of these could be perturbed, the establishment of infection could perhaps be prevented. The YxxΦ motif proximal to the membrane-spanning domain is conserved in all of the lentiviruses, and is required for the AP-2-mediated endocytosis of Env. This motif also plays a critical role in the localization of Env to the uropod [34] and polarized sorting [35,36]. Interestingly, this highly conserved motif is essential for pathogenesis in vivo, but it is not required for virus replication in vitro, at least for SIVmac. In earlier work, Hoxie, Marsh and colleagues investigated the role of the YxxΦ motif by creating a ∆GY SIVmac239 mutant, in which the Tyr of the YxxΦ motif and the adjacent Gly were deleted. The investigators studied the effect of this mutation in pig-tailed macaques, a species in which the progression to AIDS is more robust and uniform than in rhesus macaques. The infection of pig-tailed macaques with the ∆GY virus resulted in high levels of plasma viremia, but failure to deplete the mucosal CD4+ T-cells. Macrophage infection and immune activation were also not observed [37,38,39]. The animals infected with the ∆GY virus generated Abs that neutralized the parental, but not ∆GY virus. The ∆GY-infected animals displayed non-canonical CD8 responses and resisted the challenge to infection by SIVmac239. Hoxie ended his talk by discussing compensatory mutations that arose in ∆GY-infected pig-tailed macaques, in which the animals progressed to AIDS. These animals maintained ∆GY, but acquired mutations that created new YxxΦ motifs (e.g., YFQI, YEAV, or YFQL) or mutations that did not generate an identifiable YxxΦ motif (i.e., S727P, R722G, and Q738K) [37]. Marsh discussed the complex phenotype of the ∆GY mutant in the cell culture. These investigators observed lower rates of ∆GY Env endocytosis relative to WT Env, and a loss of polarized expression in Madin-Darby canine kidney (MDCK) cells, in which WT Env is largely directed to the basolateral surface. In T-cells, the ∆GY mutant showed less efficient transfer across the VS relative to WT. Some of the revertant viruses displayed a rescue in Env expression and polarized sorting [38]. Together, these data reveal significant biological properties that are influenced by the YxxΦ motif. with consequences for tissue tropism, host response, and pathogenesis.

Tom Flower (University of California, Berkeley; from Jim Hurley’s group) is developing a giant unilamellar vesicle (GUV) system to study HIV Gag lattice assembly and Env incorporation. Essential components of this experimental system, which has been used in previous studies by the Hurley lab [40,41], are myristylated and fluorescently labeled Gag, and PI(4,5)P_2_-containing GUVs. The Env was purified under the conditions developed by the Saad lab (see above and [20]). By assembling these components in vitro, Flower will be able to probe interactions between Env and the assembling Gag lattice.

In his talk, Nathan Sherer (University of Wisconsin) explored the relationship between Env and Gag recruitment to the VS, and the role of the gp41 CT and Gag MA domain in this process. To this end, Sherer’s group developed a two-color, live-cell imaging strategy to study the kinetics of Env recruitment to the VS, and the effect of Env VS localization on the recruitment of Gag to the VS [42]. This system enabled the measurement of the Env CT-dependent and MA-dependent recruitment of Gag to the VS. The CT and MA both regulated the duration and stability of the interactions between the viral donor and target cells; the 12LE mutation in MA that blocks Env incorporation [43] shortened the duration of VS formation [42]. Sherer’s findings suggest the following series of steps regulated by the Env CT and MA for cell-to-cell transmission: (1) Env–CD4 interactions between the donor and target cell, leading to VS formation, (2) MA-dependent Gag recruitment to the VS, and (3) Env CT and Gag MA maintenance of the VS during viral transmission.

Clare Jolly (University College London) continued on the theme of cell-to-cell transmission by reminding the audience that, at least in vitro, the cell-to-cell transmission of HIV-1 at a VS is much more efficient than cell-free transmission [44]. This observation is underscored by the ability of HIV-1 cell-to-cell transmission to overcome a variety of blocks to virus replication [45], including that imposed by tetherin restriction [46]. During the formation of the VS, Jolly observed significant cellular remodeling, involving the relocalization of the cell nucleus away from the VS, and the redistribution of the microtubule organizing center (MTOC), various cellular organelles, and secretory organelles towards the VS [47,48]. Because the route that Env takes to the VS is poorly understood, Jolly examined Env localization in polarized T-cells during infection and identified a ring-like concentration of Env around the MTOC proximal to the VS that was abolished by inhibiting the MTOC polarization. This finding links T-cell polarization with Env recruitment to the VS [49]. To map the dynamics of cellular signals occurring during HIV-1 cell-to-cell transmission in a mixed cell population, Jolly developed a quantitative phosphoproteomics approach [50]. This study identified over 200 host proteins modified during the HIV spread between T-cells, and found that HIV promotes virus transfer at points of cell-to-cell contact by manipulating T-cell receptor (TCR) signaling independently of antigen presentation. Related to this, the study also identified the Src-family kinase Lck as an important player in the link between cell signaling in the infected cell and viral transfer. Jolly’s group is currently extending these findings to investigate the role of signaling and host cell factors, for example Rab7a phosphorylation status, in controlling Env intracellular trafficking and delivery to viral assembly sites.

Klaus Strebel (National Institute of Allergy and Infectious Diseases, NIH) described his work on the role of human mannose receptor (hMRC1) in antagonizing lentiviral replication [51]. This factor functions in macrophages to inhibit the detachment of budded virions from the cell surface in a manner much like tetherin. Strebel’s group showed that the HIV-1_AD8_ strain counteracts the effect of hMRC1 by reducing its expression via transcriptional silencing [51]. hMRC1 directly binds all of the HIV-1 Env isolates tested, but the connection between Env and hMRC1 binding remains unclear, as the release of VLPs not bearing HIV-1 Env glycoproteins is still inhibited by hMRC1 [51]. In addition to inhibiting the virus release, hMRC1 can also reduce the particle infectivity in an Env- and strain-dependent manner. The V3 loop of gp120 appears to control the sensitivity to the loss-of-infectivity effect. The work highlights the growing number of cellular factors that interfere with the retroviral replication and the diverse array of mechanisms by which they act.

## 5. Conclusions

The 2018 International Workshop on Structure and Function of the Lentiviral gp41 Cytoplasmic Tail brought together leaders in research involving the gp41 CT to share their findings on the role of the CT in Env-related host protein interactions, structure, trafficking, virus neutralization, and transmission. Key themes that emerged during the workshop included the following: (1) the intimate relationship between MA and Env that has long been appreciated, but remains poorly understood; (2) the requirement for the CT in Env recycling and virion incorporation; (3) the role of the CT in cell-to-cell transmission in vitro, and viral dissemination and pathogenesis in vivo; and (4) novel factors involved in promoting or restricting Env function. Important questions for future research include the following: What is the structure of the gp41 CT in the context of the virus particle (i.e., with Gag present)? To what extent does particle maturation influence the structure and conformation of the CT? What similarities and differences exist between the structures of primate lentiviral gp41 CTs and the structures of other long (non-primate lentiviral) CTs vs. those of other retroviruses? Given that, despite a high variability in sequence and length, all of the retroviral Env CTs harbor motifs involved in protein trafficking, what general conclusions can be made about the role of these motifs across *Retroviridae*? Do MA and the gp41 CT interact directly, and, if so, what domains in each protein are involved? It would appear that the LLP regions of the HIV-1 gp41 CT interact with the membrane, host–cell trafficking factors, and possibly the MA domain of Gag—how are these multiple interactions choreographed and regulated?

Meeting attendees allowed for gaining new insights into the role of the gp41 CT in viral assembly, developing ideas for new techniques and research aims, discussing potential collaborations, and receiving constructive feedback on preliminary findings. This meeting achieved the primary goal of advancing the field and generating renewed energy in gp41 research. Because of the success of the meeting, it is anticipated that it will recur in a two- to three-year time frame.
